# Modelling the impact of a *Schistosoma mansoni* vaccine and mass drug administration to achieve morbidity control and transmission elimination

**DOI:** 10.1371/journal.pntd.0007349

**Published:** 2019-06-05

**Authors:** Klodeta Kura, James E. Truscott, Jaspreet Toor, Roy M. Anderson

**Affiliations:** 1 London Centre for Neglected Tropical Disease Research (LCNTDR), Department of Infectious Disease Epidemiology, School of Public Health, Faculty of Medicine, St Mary’s Campus, Imperial College London, London, United Kingdom; 2 MRC Centre for Global Infectious Disease Analysis, Department of Infectious Disease Epidemiology, School of Public Health, Faculty of Medicine, St Mary’s Campus, Imperial College London, London, United Kingdom; 3 The DeWorm3 Project, The Natural History Museum of London, London, United Kingdom; University of Washington, UNITED STATES

## Abstract

Mass drug administration (MDA) is, and has been, the principal method for the control of the schistosome helminths. Using MDA only is unlikely to eliminate the infection in areas of high transmission and the implementation of other measures such as reduced water contact improved hygiene and sanitation are required. Ideally a vaccine is needed to ensure long term benefits and eliminate the need for repeated drug treatment since infection does not seem to induce lasting protective immunity. Currently, a candidate vaccine is under trial in a baboon animal model, and very encouraging results have been reported. In this paper, we develop an individual-based stochastic model to evaluate the effect of a vaccine with similar properties in humans to those recorded in baboons in achieving the World Health Organization (WHO) goals of morbidity control and elimination as a public health problem in populations living in a variety of transmission settings. MDA and vaccination assuming different durations of protection and coverage levels, alone or in combination, are examined as treatment strategies to reach the WHO goals of the elimination of morbidity and mortality in the coming decade. We find that the efficacy of a vaccine as an adjunct or main control tool will depend critically on a number of factors including the average duration of protection it provides, vaccine efficacy and the baseline prevalence prior to immunization. In low prevalence settings, simulations suggest that the WHO goals can be achieved for all treatment strategies. In moderate prevalence settings, a vaccine that provides 5 years of protection, can achieve both goals within 15 years of treatment. In high prevalence settings, by vaccinating at age 1, 6 and 11 we can achieve the morbidity control with a probability of nearly 0.89 but we cannot achieve elimination as a public health problem goal. A combined vaccination and MDA treatment plan has the greatest chance of achieving the WHO goals in the shorter term.

## Author summary

Nearly 258 million people are infected worldwide by schistosome parasites. The World Health Organization (WHO) has set control guidelines to combat the morbidity and mortality induced by infection, defined by reaching ≤5% and ≤1% prevalence of heavy-intensity infections in school-aged children (SAC), respectively. Mass drug administration (MDA) is the major route for morbidity control and elimination. However, MDA does not provide long-term protection against schistosome parasites and frequent drug administration is therefore required to control morbidity. Infection does not induce lasting acquired immunity to reinfection. Drug resistance is another issue with MDA which, if it arises, could possibly make drug treatment ineffective over time as drug-resistant genes in the parasite population increase in frequency. A vaccine is ideally needed to both reduce the possibility of reinfection and to achieve transmission elimination within a feasible time frame. Based on the recent results obtained for a new candidate vaccine in the baboon animal model, we employ an individual-based stochastic model to assess the impact of a vaccine with an efficacy of 100% when applied in endemic regions with different intensities of transmission. Simulations suggest that the probability of achieving morbidity control and elimination as a public health problem depends on the duration of protection provided by vaccination, the age categories of the human host population vaccinated, and the coverage levels achieved. In order to achieve elimination as a public health problem, model simulations suggest that combining vaccination (with 5 years of protection) with MDA (treating 75% of school-aged children, 5–14 years of age) is the best option, particularly in high transmission settings.

## Introduction

Schistosomiasis inflicts significant levels of human morbidity and mortality in regions of the world with endemic infection. It is estimated that nearly 258 million people are infected worldwide with up to 700 million at risk of being infected, leading to an estimated 280000 deaths annually [1–3]. Schistosomiasis is an intestinal or urogenital disease caused predominantly by infection with *Schistosoma mansoni*, *S*. *japonicum* or *S*. *haematobium*, and is one of the diseases included within the World Health Organization (WHO) 2020 goals for neglected tropical diseases (NTD) control. Individuals become infected when cercariae (larval forms of the parasitic worm), released by an intermediate host (various freshwater snail species), penetrate the skin during contact with contaminated water [4]. Control programmes are at present based on mass drug administration (MDA) using the drug praziquantel, and behaviour modification directed at reducing water contact and improvements in sanitation. MDA has to be repeatedly used, since clearing infection does not result in acquired immunity and treated individuals can be re-infected. Age-related water contact behaviour results in most infection residing in school-aged children (SAC; 5–14 years of age), since age intensity of infection profiles are convex in shape. Treatment is therefore specifically focused on this age group. At present, pre-school aged children (pre-SAC) are not eligible for treatment with praziquantel [5] due to the absence of clinical data on the drug effects and safety in the very young. In the coming years a new formulation of praziquantel may be approved for very young children [6]. In areas of high transmission, WHO guidelines also recommend treatment of adults at risk [1], [7]. By 2020, WHO aims to increase coverage in areas of endemic infection such that 75% of SAC at risk will be regularly treated [2], but progress to date in reaching this target has been poor in many regions.

Currently WHO recommends using prevalence of infection in SAC to determine how often to treat in a given endemic area [1]. The recommended treatment strategy for schistosome infection is dependent upon whether the community has a low (< 10%), moderate (10–50%) or high (≥ 50%) prevalence at baseline before the implementation of MDA. The strategy for low-risk communities is to treat all SAC twice during their primary schooling age, generally once every three years, and supply praziquantel in local health centres to treat suspected cases. For moderate-risk communities, the recommendation is to treat all SAC and at-risk adults once every two years. For high-risk communities, the recommended approach is to treat all SAC and at-risk adults once a year. At present in national NTD control programmes, schistosomiasis has one of the lowest levels of MDA coverage of all helminth diseases [8], [9].

Given that MDA needs to be administered to individuals frequently, and that it does not provide long-term protection against the infection in the absence of a strong acquired immunological response to infection, a vaccine is ideally needed for control in the longer term. At present, there is no vaccine for use in humans that can protect against the schistosome infection. However, recent experimental studies by Afzal Siddiqui and colleagues on a candidate vaccine against *Schistosoma mansoni* infection in a baboon animal model have produced some encouraging results. In four independent, double-blinded studies, a Sm-p80-based vaccine exhibited potent prophylactic, anti-egg induced pathology and transmission-blocking efficacy against *S*. *mansoni* in the baboon (*Papio ursinus*) animal model [10]. The vaccine reduced female worm establishment by 93.45% and significantly resolved the major clinical manifestations of hepatic/intestinal schistosomiasis by reducing the tissue-egg load by 91.35%. A 40-fold decrease in faecal egg excretion by those few female parasites that established in the vaccinated animals, combined with a 79.21% reduction in hatching ability of eggs (the release of viable miracidia), suggests the vaccine may have a high transmission blocking potential. The study showed comprehensive evidence for the effectiveness of a Sm-p80-based vaccine for schistosomiasis and provided support for the need to move beyond animal models to human studies.

Based on the baboon experiments by Siddiqui and colleagues, and assuming efficacy would be similar in humans, published epidemiological analyses based on mathematical models have predicted that the Sm-p80-based vaccine could potentially block infection in areas of low and moderate transmission provided the duration of protection provided by the vaccine is 5 years or more [11], [12]. These models were simple in structure and built on a deterministic framework. This study extends these analyses using an individual based stochastic model to look at the impact of a vaccine, with varying durations of protection, employed in different community-based vaccination programmes involving either vaccinating young children in a cohort-based approach or vaccinating the whole community across all age classes). Analyses are also presented of the impact on transmission and the prevailing levels of infection using either vaccination alone, MDA alone (the current most commonly used intervention to control morbidity) and or using both in different combinations. A description of the impact of MDA, alone on the prevalence and intensity of *S*. *mansoni* infection in various transmission settings, is covered in a series of recent publications, as is model structure, model assumptions and data sources for the key transmission and biological parameters [3], [4], [7], [8].

The focus in the present analyses is on the relative merits of vaccination versus MDA, alone or in combination, as a tool for the community control of the morbidity induced by *S*. *mansoni* and the likelihood of transmission elimination.

## Methods

### The model

Past work on the impact of MDA on *Schistosoma mansoni* has employed a hybrid deterministic model (with deterministic and stochastic components) based on sets of partial-differential equations to describe changes in the mean worm burden *M*(*t*, *a*), for host *a* over time *t* [13–15].

Stylianou et al, developed an age independent deterministic model to explore the effect of community vaccination programmes [11]. We extend this deterministic model and develop an individual-based stochastic model (an earlier version is described in [4]), where an individual of age *a* can be in one of the two categories; (i) unvaccinated group or (ii) vaccinated group, denoted by *N*_*u*_(*a*, *t*) and *N*_*v*_(*a*, *t*) respectively. We assume that the number of births is the same as the number of deaths (constant size for the human host), hence the total population of age *a*, at time *t* is *N*(*a*, *t*) = *N*_*u*_(*a*, *t*) +*N*_*v*_(*a*, *t*). The unvaccinated and vaccinated host dynamics can be described by the following system of partial differential equations (PDEs):

$$\frac{\partial N_{u}(a,t)}{\partial t}+\frac{\partial N_{u}(a,t)}{\partial a}=-q(a,t)N_{u}(a,t)+\omega N_{v}(a,t)-\mu (a)N_{u}(a,t)$$ (1)

$$\frac{\partial N_{v}(a,t)}{\partial t}+\frac{\partial N_{v}(a,t)}{\partial a}=q(a,t)N_{u}(a,t)-\omega N_{v}(a,t)-\mu (a)N_{v}(a,t)$$ (2)

Here *q*(*a*,*t*) is the fraction of the population of age *a* vaccinated at time *t*, $\omega =\frac{1}{\text{duration}\;\text{of}\;\text{vaccine}\;\text{protection}}$ is the vaccine decay rate and *μ*(*a*) is the host mortality rate.

The vaccine candidate is assumed to act on the following variables [cf. Eqs (3) and (4)]; (i) parasite establishment within the human host by reducing the rate of infection, *β*, (ii) parasite survival and growth within the human host, by reducing adult worm life expectancy, *σ* and (iii) reducing the rate of egg production, *λ*, due to a reduced growth rate in humans. We assume that the vaccine’s impact on worm death rate, eggs per gram (EPG) and age-specific contact rates are *v*_1_, *v*_2_ and *v*_3_ respectively, where the values range from 0 to 1. The total worm burden in the unvaccinated and vaccinated hosts are denoted by *M*_*u*_ and *M*_*v*_ and the changes in *M*_*u*_ and *M*_*v*_, over time for host *a* are described by the following equations:

$$\frac{\partial M_{u}(a,t)}{\partial t}+\frac{\partial M_{u}(a,t)}{\partial a}=L\beta (a)N_{u}(a,t)-q(a,t)M_{u}(a,t)+\omega M_{v}(a,t)-(\mu (a)+\sigma )M_{u}(a,t)$$ (3)

$$\frac{\partial M_{v}(a,t)}{\partial t}+\frac{\partial M_{v}(a,t)}{\partial a}=Lv_{3}\beta (a)N_{v}(a,t)+q(a,t)M_{u}(a,t)-\omega M_{v}(a,t)-(\mu (a)+v_{1}\sigma )M_{v}(a,t)$$ (4)

Here *L* represents the concentration of the infectious material in the environment, namely, how each individual of age *a*, contributes to the pool of released eggs. This is discussed in detail in [14] and [16]. It is assumed that the rates of turn over for the miracidia, snail intermediate host and cercaria are much faster (life expectancies days to weeks) than the adult worm in the human host (life expectancy 4–6 years), so the dynamics of these life cycle stages are collapsed into the equations for the adult worms in humans as detailed in Anderson & May [15].

The total worm burden in the population is given by the sum of the total worm burden in the unvaccinated and vaccinated hosts.

If we denote the total worm burden in the population as the sum of the total worm burden in the unvaccinated and vaccinated hosts by $\bar{M(a,t)}=M_{u}(a,t)+M_{v}(a,t)$ and add Eqs (3) and (4) together we obtain the following,

$$\frac{\partial \bar{M(a,t)}}{\partial t}+\frac{\partial \bar{M(a,t)}}{\partial a}=L\beta (a)N_{u}(a,t)+Lv_{3}\beta (a)N_{v}(a,t)-\sigma \bar{M(a,t)}-\mu (a)\bar{M(a,t)}$$ (5)

In Eq (5) we have assumed *v*_1_ = 1. We can express $\bar{M(a,t)}$ in terms of the mean worm burden, *M*(*a*,*t*), as $\bar{M(a,t)}=N(a,t)M(a,t)$. Then we obtain;

$$\frac{\partial M(t,a)}{\partial t}+\frac{\partial M(t,a)}{\partial a}=\frac{Lv_{3}\beta (a)N_{v}(a,t)+L\beta (a)N_{u}(a,t)}{N(a,t)}-\sigma M(a,t)$$ (6)

The egg output (from the vaccinated and unvaccinated populations) is given by

$$E=\frac{\psi }{\bar{L}}\int_{a=0}^{\infty }\left\{N_{u}(a,t)F(\frac{M_{u}(a,t)}{N_{u}(a,t)};\lambda )+N_{v}(a,t)F(\frac{M_{v}(a,t)}{N_{v}(a,t)};v_{2}\lambda )\right\}\rho (a)da$$ (7)

given

$$\frac{dL}{dt}=E-\mu_{2}L$$ (8)

where the death rate is that of infected snails.

In the above equation *ψ* describes the flow of the infectious material into the reservoir while the function *F*(*M*(*a*,*t*); *λ*) generates the egg output as a function of mean worm burden and *ρ*(*a*) represents the age-specific relative contribution of infectious stages to the environmental reservoir. In our simulations we assume the host contribution to the reservoir to be the same as the age-specific contact rates, *β*(*a*).

This model has a full age structure for the human host where the outputs are grouped into three age categories, pre-SAC (0–4 years of age), SAC (5–14 years of age) and adults (15+ years of age). We use these age groupings based on WHO definitions of treatment groups [1–3] to calculate the necessary coverage levels (MDA or vaccination) for each category in order to interrupt transmission. This is typically defined as the overall *R*_0_ <1 in infectious disease epidemiology, but as shown by Anderson and May [14], the system of equations defined above has three possible equilibria; namely, a stable endemic state, an unstable boundary (transmission breakpoint) and a stable state of parasite extinction. This model is hybrid in the sense that assumes a negative binomial form for the distribution of parasite numbers per host with a fixed aggregation parameter *k*, density dependent fecundity, and assumed monogamous sexual reproduction among worms.

The mean expected behavior of the individual based stochastic model is identical to the predictions of a deterministic version of the model. However, an individual-based stochastic model permits the examination of the probability distribution of a given event occurring, such as transmission elimination, in a defined period of time during which control measures are applied.

Autopsy data show that worms tend to aggregate more in some individuals than in others, due to poorly understood factors such as environmental, social, host genetic or immunological effects [17]. Epidemiological studies also show that those heavily infected are predisposed to this state [18]. To take account of such effects in our model, individuals in each age category are assigned a contact rate drawn from a gamma distribution with shape parameter *α*, which, via compounding across individual distributions, leads to a negative binomial distribution of worms within the total host population. It is important to note that the aggregation parameter, *k*, within the stochastic model, fluctuates in value over time, as a result of changes in the mean worm burden. In the deterministic model k is held fixed in value. The stochastic model more accurately mirrors observed patterns where k tends to decrease in value as prevalence declines under the impact of control measures [19]. The egg contribution to the infectious reservoir depends on the age-specific contact rate for each individual and is governed by a deterministic formulation. Treatment events are predetermined, they occur at time *t*_*j*_ and the time step to the next treatment event is randomly drawn from an exponential distribution. The rate parameter for this distribution is given by the overall rate that any event happens. Which event occurs is drawn at random, on the basis of the relative magnitude of each individual event relative to the combined rate of all events. Table 1 provides a description of these rates. In this paper we consider 15 years of MDA and vaccination administration.

**Table 1:** Table of events for the stochastic model (as in [16]), where *λ*_*i*_ is the gamma distribution for individual *i*, *δ*() is the Dirac delta function and *g* is the proportion treated.

Event	Rate
Per capita worm acquisition by host *i*, aged *a*, per unit of time	*v*_3_*β*(*a*_*i*_)*v*_2_*λ*_*i*_*L* per host per unit of time
Worm death in host *i* per year per unit of time	*v*_1_*σ* per worm per unit of time
Host birth/death for host aged *a* years	*μ*(*a*_*i*_) per unit of time
Treatment of host *i*, aged *a* years	*δ*(*t* − *t*_*j*_)*g*(*a*_*i*_)

Most of the parameter values used in this paper are taken from within the ranges found in the literature (Table 2). However, the data for the age-specific contact rates of hosts within the infectious reservoir (β) and age-specific contribution of hosts to the reservoir are unknown. They are estimated by using MCMC method in parameter estimation from age intensity and prevalence curves as described in references detailed in the text and Table 2. Precise details of the model fitting procedure are described in previous publications [4,14,15,17].

**Table 2:** Parameter definition and age specific contact rates for *S*.*mansoni* in Iietune, Kenya [3], [4].

Parameter	Value	Source
Population size	500	-
Fecundity (λ)	0.14 eggs/female/sample	[4]
Aggregation parameter (k)	0.04 in low settings;0.24 in moderate and high settings	[4], [20]
Worm lifespan	5.7 years	[3], [13], [21]
Drug efficacy	86.3%	[22]
Impact of vaccine on worm death rate (***v***_**1**_)	1	-
Impact of vaccine on eggs per gram (***v***_**2**_)	0	-
Impact of vaccine on contact rates (***v***_**3**_)	0	-
Age specific contact rates (***β***)	For 0–4, 5–9, 10–15, 16+ years of age: 0.032, 0.162, 1, 0.06	[3], [4]
Contribution to the reservoir by contact age group (***ρ***)	For 0–4, 5–9, 10–15, 16+ years of age: 0.032, 0.162, 1, 0.06	[3], [4]
SAC prevalence (%)	SAC having egg count threshold > 0	-
SAC Heavy-intensity infection prevalence (%)	SAC having egg count threshold > 16	[23]
High baseline prevalence (≥ **50%**)	*R*_0_ = 3.2–5	-
Moderate baseline prevalence (10–50%)	*R*_0_ = 1.8	-
Low baseline prevalence (< **10%**)	*R*_0_ = 1.7	-

### MDA and vaccine treatment

In the numerical evaluations of the model’s behavior (stochastic simulations), we follow the WHO guidelines for the implementation of MDA. Starting with an untreated population, we administrate MDA over a 15-year period with coverage levels and treatment intervals based on the baseline prevalence. For low baseline prevalence in SAC, we treat once every 3 years; for moderate baseline prevalence in SAC, we treat once every 2 years and for high baseline prevalence in SAC, we treat once a year. The intensity of transmission is determined by *R*_0_ (the basic reproductive number) which varies for different baseline settings. When MDA alone is used as the treatment strategy, we simulate the following treatment strategies: (i) the WHO recommended treatment coverage of 75% SAC only; (ii) 60% of SAC only; (iii) 40% of SAC only and (iv) 85% of SAC and 40% of adults.

In this paper we consider an ideal case-perfect vaccine, meaning that the rate of infection and the rate of egg production are essentially reduced by 100%, which is comparable to the efficacy of the Sm-p80 vaccine in the baboon model. This efficacy considers the prevention of worm establishment, the fecundity falling dramatically in those few worms that establish, and the inability of eggs from these worms to hatch and release viable miracidia. Vaccination is given annually to the children with the pre-specified age of administration, and the coverage levels depend on the age group that is treated and the duration of vaccine protection. In various experimental settings Sm-p80 has demonstrated robust antibody titres in baboons for up to 5–8 years [10] suggesting a reasonably long duration of protection. In this paper we simulate scenarios where (i) the vaccine gives a 5 year duration of protection (from [10]) and (ii) an ideal scenario where the vaccine gives a 20 years of protection which is longer than the duration of treatment (15 years). It should be noted here that the same results will be obtained for vaccines with a duration of protection longer than 20 years as we are only calculating the probability of achieving the WHO goals within 15 years of initiating vaccination. Also, it should be noted that the vaccine decay rate is given by 1/ (duration of protection). Duration of vaccine protection has a direct impact on the vaccine administration schedule and the coverage levels required to have a significant impact. Here we consider the epidemiology of schistosome infections and the human host age-groups contributing most to parasite transmission. The aim is to cover children from ages 5–15 by vaccinating children in cohorts. We also analyze control strategies where the vaccine is given to younger children in their first year of life. The schistosomiasis vaccine will very likely be administered in conjunction with other vaccines already present in traditional immunization programmes (HPV, DTP). Therefore, the achievable coverage will typically match that achieved for one of the other co-administered vaccines. Vaccination coverage in the first year of life ranges between 85% and 91% at global level and reduces significantly in the following years (Table 3). The coverage levels for school age children vary between 60% and 70% and for out of school individuals this range is 40%-50% [24–27].

**Table 3:** Vaccination coverage achieved for HPV and DTP.

Age group	Coverage level	Source
1-year olds	85%-91%	[27]
SAC (5–14)	60%-70%	[24–26]
Adults (15+)	40%-50%	[24–26]

Based on these coverage levels, for a vaccine that provides a 20-year protection against schistosomiasis, we vaccinate at age 1 (early start) or age 5 (school start), with coverage levels of 85% and 60% respectively. For a vaccine that provides a 5-year duration of protection against infection, to ensure continuous protection, we vaccinate either at ages 1, 6 and 11 with coverage levels 85%, 60% and 70% respectively, or at ages 5,10 and 15 with coverage levels 60%, 70% and 45% respectively. In this case (5-year duration of protection) we have a 3-dose schedule of vaccination, similar to the HPV administration schedule.

We consider MDA and vaccination, alone or in combination, as control strategies, where treatment is delivered at random at each round within the population with a given coverage. In other words, we do not consider individual compliance to treatment [19] in these analyses and just assume the individuals treated or vaccinated are chosen at random at each round.

**Fig 1:**
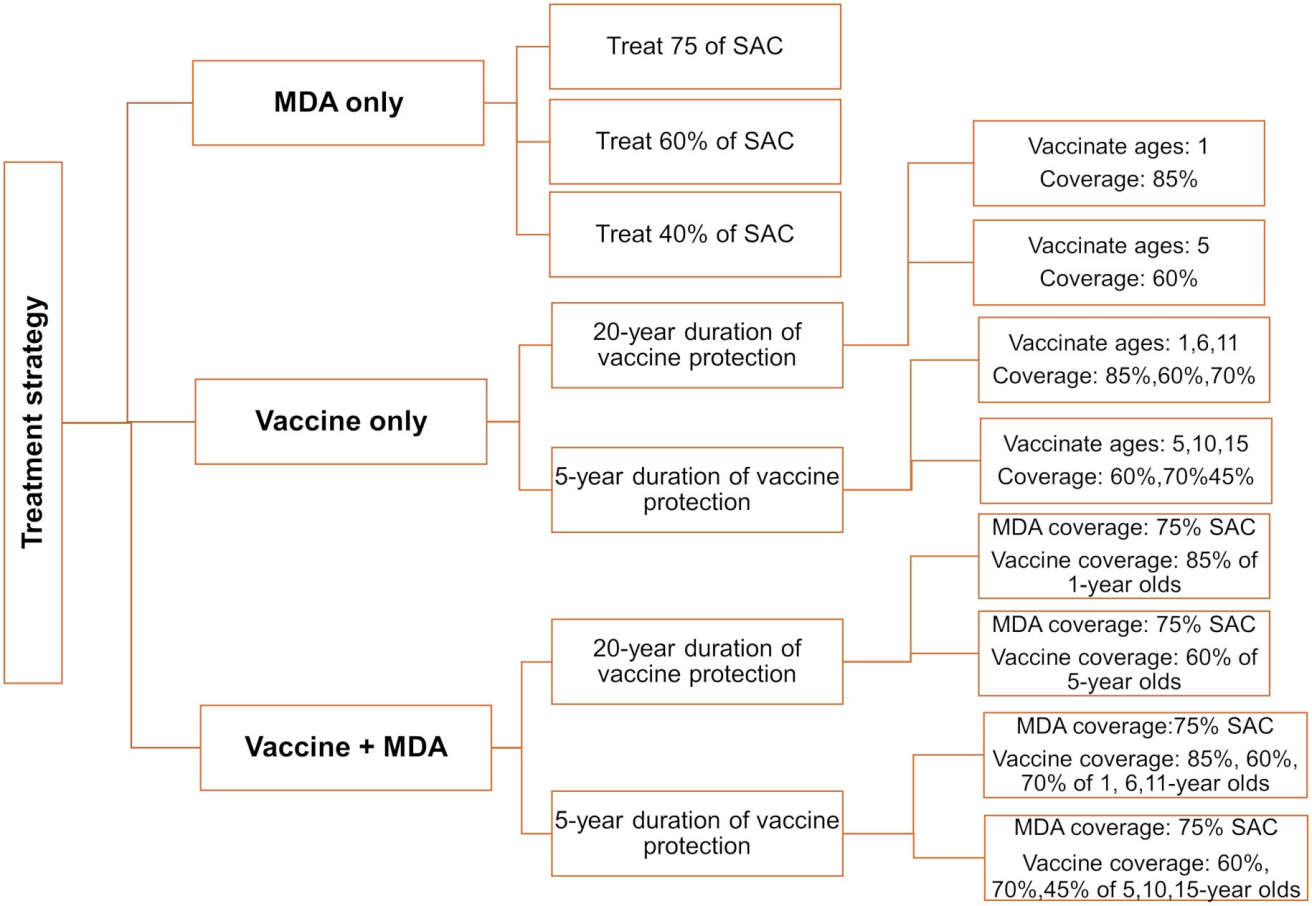
Coverage levels for the infected population. Treatment strategies examined in the simulations for different transmission settings.

At the end of the treatment period, we calculate the probability of reaching WHO morbidity and elimination as a public health problem goal, by evaluating the fraction of SAC heavy-intensity infection prevalence (≤5% heavy-intensity infection in SAC for the morbidity goal and ≤1% heavy-intensity infection in SAC for the elimination as a public health problem goal). In our results we include the prevalence of infection (population having egg count threshold > 0) and prevalence of heavy-intensity infections (population having egg count threshold > 16). The probability of reaching the 5% and 1% WHO goals are calculated as the fraction of repetitions that reach the target, by averaging across 300 simulations (to ascertain the mean expectation of the stochastic model). A summary of the treatment strategies is presented in Fig 1.

## Results

In presenting the results of the stochastic model simulations for the various scenarios described above, the impact of the candidate vaccine and/or MDA is depicted by reference to the prevalence and mean intensity of *S*. *mansoni* infection in low, moderate and high transmission settings. For each treatment strategy, the prevalence of infection and prevalence of heavy-intensity infections in SAC and adults (the morbidity goal set by WHO), as well as the probability of achieving the WHO goals at all times *t*, until *t* = 15 (the end of control interventions), are assessed.

### MDA alone: Treating SAC only

**Fig 2:**
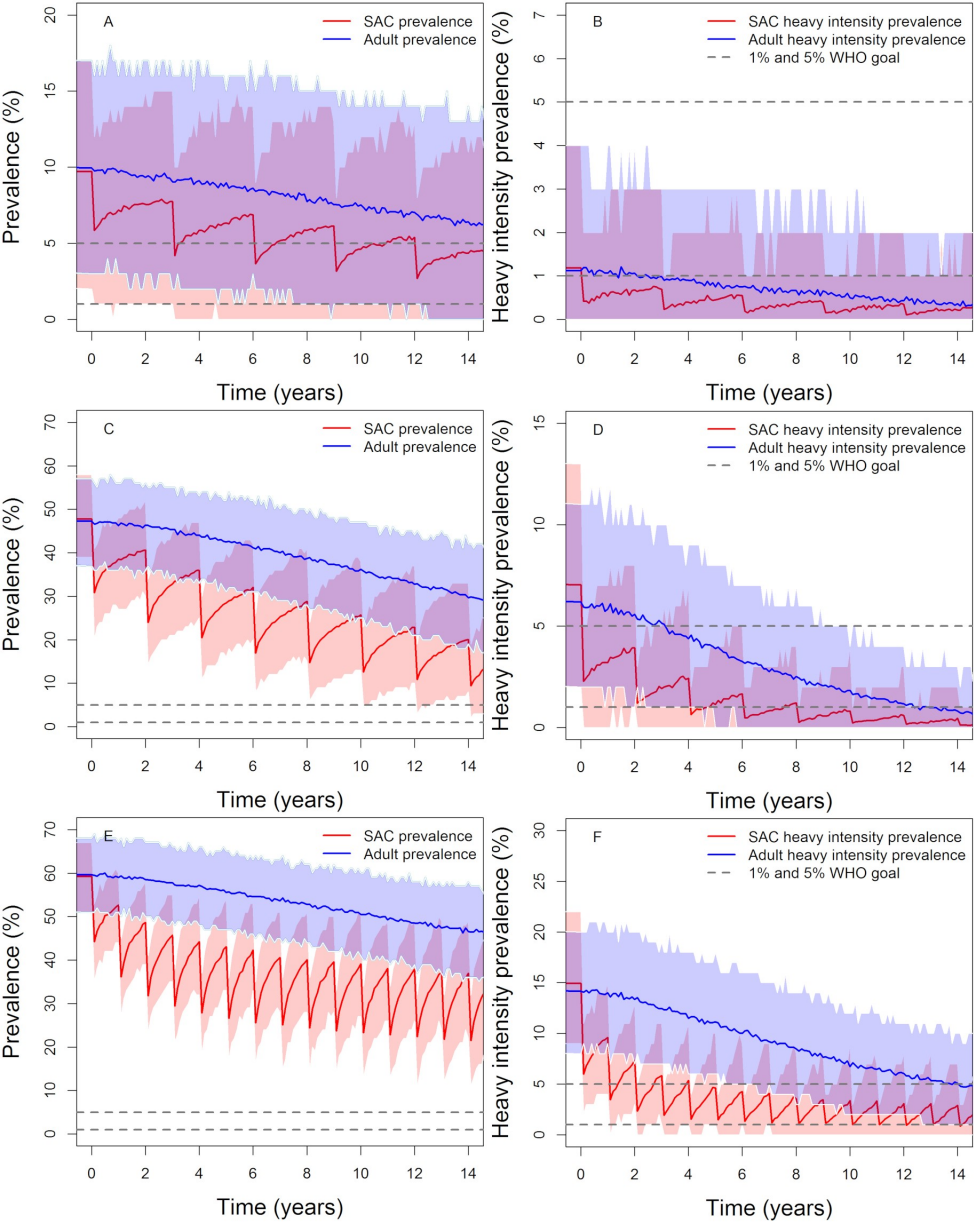
Model projections of MDA treatment of 75% school-aged children (SAC; 5–14 years of age) in low (first row), moderate (second row) and high (thrird row) baseline transmission settings. Graphs A, C and E show the prevalence of infection, Graphs B, D and F show the prevalence of heavy-intensity infections in school-aged children (SAC) and adults. The WHO target of 75% SAC coverage is assumed from the start of treatment programme throughout the 15 years of treatment. Treating annually (low settings) or twice a year (moderate settings) reaches the 5% morbidity and 1% elimination as a public health problem goals by year 15. Shaded areas (both blue and red) represent the 90% credible interval (90% of the simulated results fall within these shaded areas).

First MDA alone is examined as the treatment strategy, using the WHO targets for treatment of 75% coverage for SAC. The results are presented in Fig 2 and Table 4. Model simulations (based on the parameter values listed in Table 2) suggest that for low prevalence regions, the 5% morbidity goal in SAC can be achieved within 5 years of treatment, while the elimination as a public health problem goal in the total population can be achieved within 10 years of treatment.

**Table 4:** Probability of reaching the WHO 5% morbidity control and 1% elimination as a public health problem goals using MDA only, treating once every 3 years in low transmission settings, once every 2 years in moderate transmission settings and once a year in high transmission settings. Green shaded areas = probability of reaching target > 0.9, Yellow shaded areas = 0.5 ≤ probability of reaching target < 0.9, Red shaded areas = probability of reaching target <0.5.

Transmission setting	Coverage	Probability of achieving morbidity control (≤5% heavy infection in SAC)			Probability of achieving elimination as a public health problem (≤1% heavy infection in SAC)		
		Year 5	Year 10	Year 15	Year 5	Year 10	Year 15
**Low**	**75% SAC**	1	1	1	0.870	0.963	0.970
	**60% SAC**	1	1	1	0.850	0.893	0.923
	**40% SAC**	0.987	0.993	0.993	0.790	0.873	0.840
**Moderate**	**75% SAC**	0.990	0.993	1	0.450	0.780	0.960
	**60% SAC**	0.937	0.963	1	0.350	0.580	0.720
	**40% SAC**	0.720	0.833	0.900	0.190	0.273	0.400
**High**	**75% SAC**	0.670	0.800	0.850	0.100	0.250	0.350
	**60% SAC**	0.340	0.540	0.620	0.400	0.600	0.110
	**40% SAC**	0.180	0.200	0.210	0.000	0.000	0.100

Similarly, for moderate-prevalence regions, the 5% morbidity goal in SAC can be achieved within 5 years of treatment, whereas the 1% elimination as a public health problem goal can be achieved within 15 year of MDA treatment. Again, both goals will be achieved within 15 years with a probability of unity.

In high transmission regions, we can achieve the SAC 5% morbidity goal in 85% of the simulations. However, the 1% elimination as a public health problem goal in such high transmission (large R_0_ values) settings can be achieved in 35% of our simulations. In these settings, increasing the SAC coverage to > 75% and/or include other age bands in the treatment is highly desirable.

In low to moderate transmission settings, using the recommended target coverage of 75% for SAC, the SAC 5% morbidity goal can be achieved within 5 years of MDA. Given the difficulties countries with endemic infection are experiencing in achieving this level of coverage, SAC coverages between 40% and 60% were also examined to explore if it is still possible to achieve the WHO goals with 15 years of MDA treatment.

The impact of MDA decreases as SAC coverage declines as indicated in Table 4. The SAC 5% morbidity goal can be achieved within 5 years at 60% SAC coverage (in low to moderate settings). However, for the <1% heavy infection in the total population goal (= elimination as a public health problem) to be achieved within 15 years the probabilities of achieving this are 90% and 70%, respectively, in low and moderate transmission regions.

Lowering the SAC coverage to 40% is predicted to achieve the WHO goals in low transmission settings. However, in moderate transmission settings, the SAC 5% morbidity goal can be achieved within 15 years of treatment with probability of 0.9, but the 1% elimination as a public health problem goal is only achieved with probability 0.4 in that time. These results highlight the importance of using different MDA coverage levels in different transmission settings, as opposed to following the recommended 75% SAC coverage for all transmission levels.

In stochastic (and deterministic) models (and in the real world) there is always a chance that the prevalence of infection will bounce back after control measures cease since in some simulation runs the breakpoint in transmission is not crossed. It is therefore important to analyze the probability of true elimination (also known as ‘transmission interruption’) which results in the prevalence within the whole community in which control measures are introduced going to zero. As in previous studies [28] it is assumed that if the overall prevalence is less than 1% it is almost certain that transmission interruption has been achieved. We find that treating only 75% of SAC cannot interrupt transmission (see Fig 2A, 2C and 2E), since the reservoir of untreated people in the adult age classes is able to seed the whole population once control ceases at year 15.

### MDA alone: Treating SAC and adults

**Fig 3:**
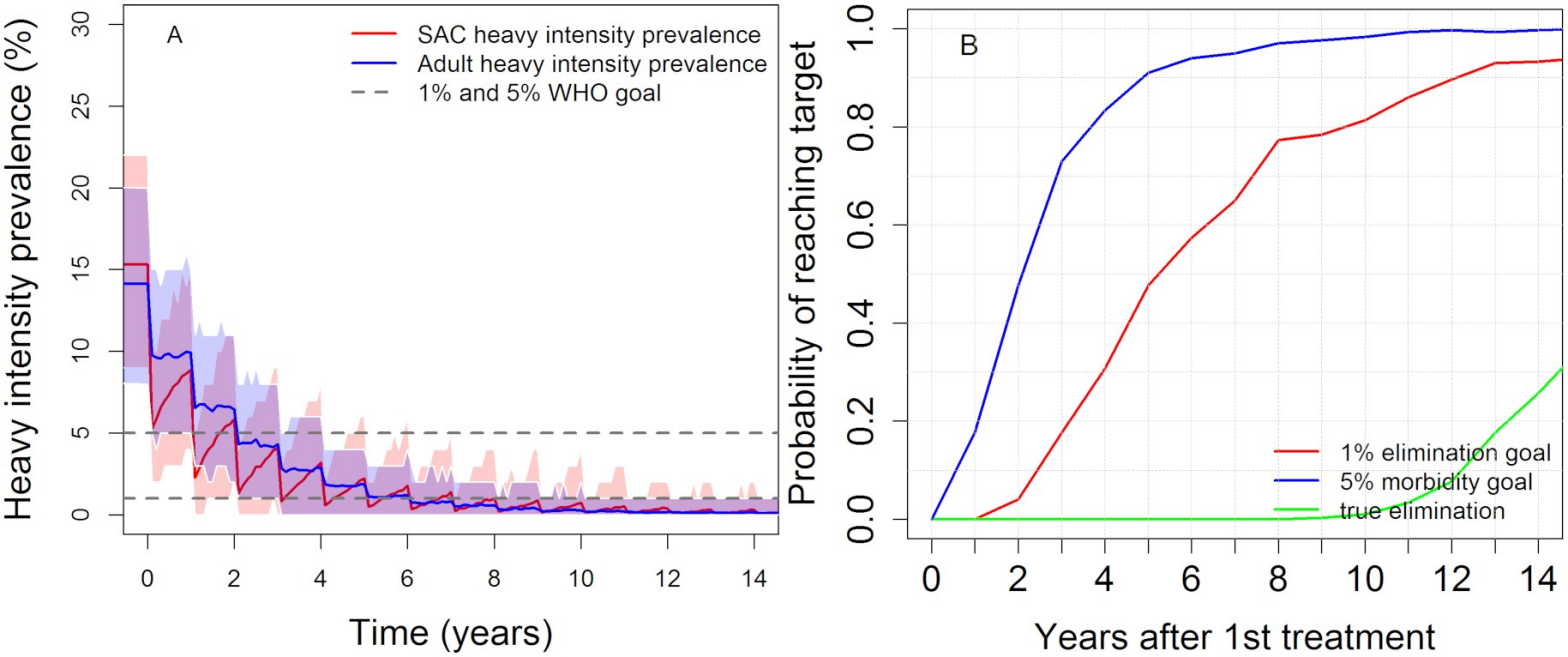
Model projections of annual MDA treatment of 85% school-aged children (SAC; 5–14 years of age) and 40% adults (15+ years of age). Shown for high prevalence settings (≥50% SAC baseline prevalence). The shaded areas (both blue and red) represent the 90% credible interval. Graph (A) shows the prevalence of heavy-intensity infections in school-aged children (SAC) and adults. Graph (B) represents the probability of reaching the WHO goals by year 15.

As discussed earlier, in high transmission settings it is necessary to treat both SAC and adults. Here we present the simulation results for the scenario 85% of SAC and 40% of adults are annually treated with MDA. These results are summarized in Fig 3 which shows that with this approach the WHO goals can be achieved, although the probability of complete elimination by year 15 is still low (<0.3). Longer durations of treatment and/or more frequent treatment are required to increase this probability.

### Vaccination alone: Treating pre-school and school aged children

In this section, the effects of both vaccination coverage, and the average duration of protection provided by the vaccine, are examined. It should be noted that, based on the animal model results, we assume the vaccine is 100% efficacious.

#### Duration of vaccine protection = 20 years

**Fig 4:**
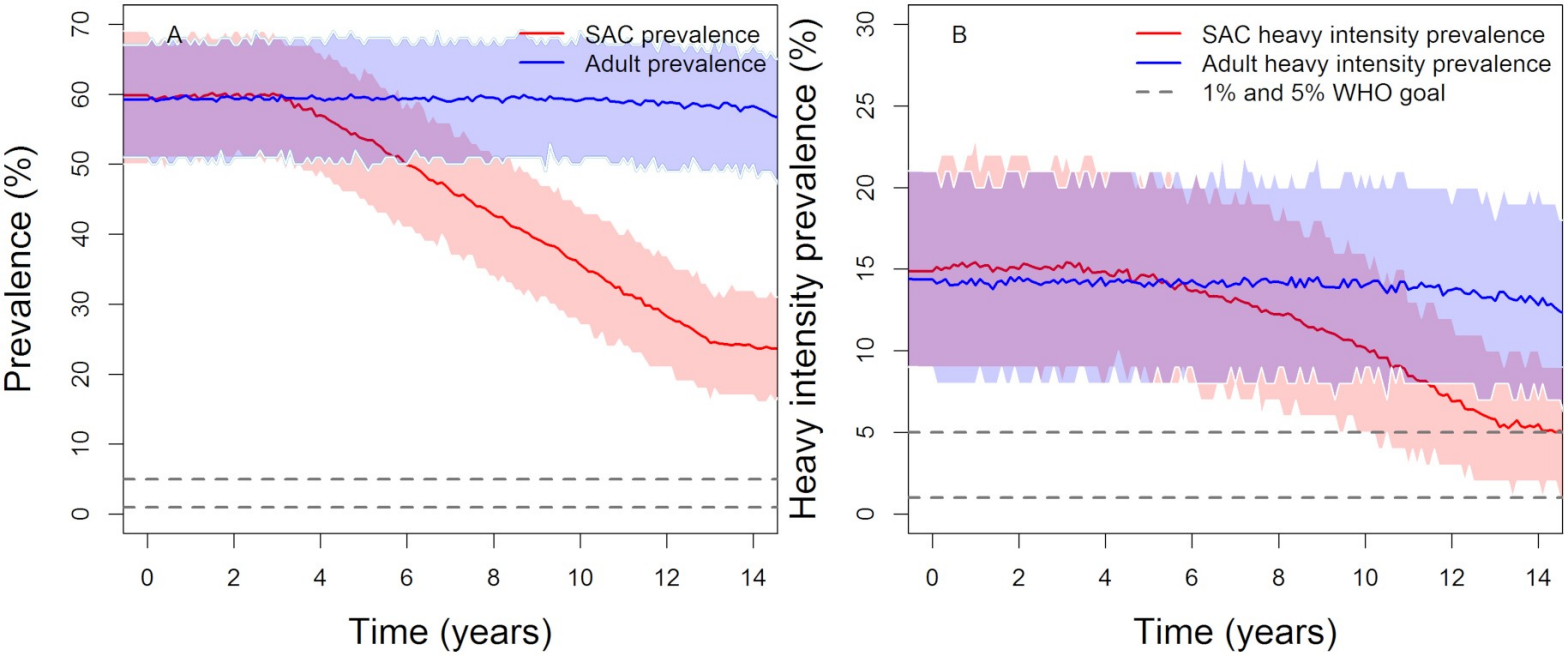
Model projections of annual vaccination of 85% of 1 year olds in high baseline transmission settings. Graph (A) shows the prevalence of infection, Graph (B) shows the prevalence of heavy-intensity infections, both in school-aged children (SAC) and adults. The average duration of vaccine protection is set at 20 years. Shaded areas (both blue and red) represent the 90% credible interval (90% of the simulated results fall within these shaded areas).

In the case where the vaccine gives on average 20-years of full protection (a very long duration) against *S*. *mansoni* infection, we consider two treatment strategies; namely: (i) pre-school vaccination (early start) by vaccinating 85% of 1-year olds or (ii) school-age children vaccination where 60% of 5-year olds (school-start) are vaccinated. The stochastic model predicts the following outcomes for strategy (i) and (ii) in part displayed in Fig 4 and Table 5.

1. In low transmission settings, for both control strategies, the SAC 5% morbidity goal can be achieved within 5 years of young cohort vaccination and the 1% elimination as a public health problem goal can be achieved within 15 years of vaccination. Both events occur with a probability of nearly 1.
2. In moderate transmission settings, for both control strategies, the 1% elimination as a public health problem goal can only be achieved with a probability of roughly 0.5 at year 15. However, the SAC 5% morbidity control goal can be achieved with a probability of 0.98 at year 15.
3. In high transmission settings and late vaccination at 5 years of age, the 1% elimination as a public health problem goal will rarely be achieved. Treating at age 1, however, can achieve the 5% morbidity goal with a probability of approximately 0.61. If we treat at age 5, the simulations suggest that we can achieve this goal with a probability of approximately 0.55.

**Table 5:** Probability of reaching the WHO 1% elimination as a public health problem goal and the 5% SAC 5% morbidity goal for vaccination only with different coverage levels. The duration of vaccine induced protection is set at 20 years. Green shaded areas = probability of reaching target > 0.9, Yellow shaded areas = 0.5 ≤ probability of reaching target < 0.9, Red shaded areas = probability of reaching target <0.5.

Transmission setting	Coverage	Probability of achieving morbidity control (≤5% heavy infection in SAC)			Probability of achieving elimination as a public health problem (≤1% heavy infection in SAC)		
		Year 5	Year 10	Year 15	Year 5	Year 10	Year 15
**Low**	**85% at age 1**	0.990	0.997	1	0.760	0.840	0.953
	**60% at age 5**	1	1	1	0.780	0.920	0.980
**Moderate**	**85% at age 1**	0.413	0.570	0.980	0.027	0.053	0.523
	**60% at age 5**	0.383	0.900	0.987	0.037	0.283	0.553
**High**	**85% at age 1**	0.000	0.090	0.610	0.000	0.000	0.060
	**60% at age 5**	0.100	0.340	0.550	0.000	0.000	0.020

#### Duration of vaccine protection = 5 years

**Fig 5:**
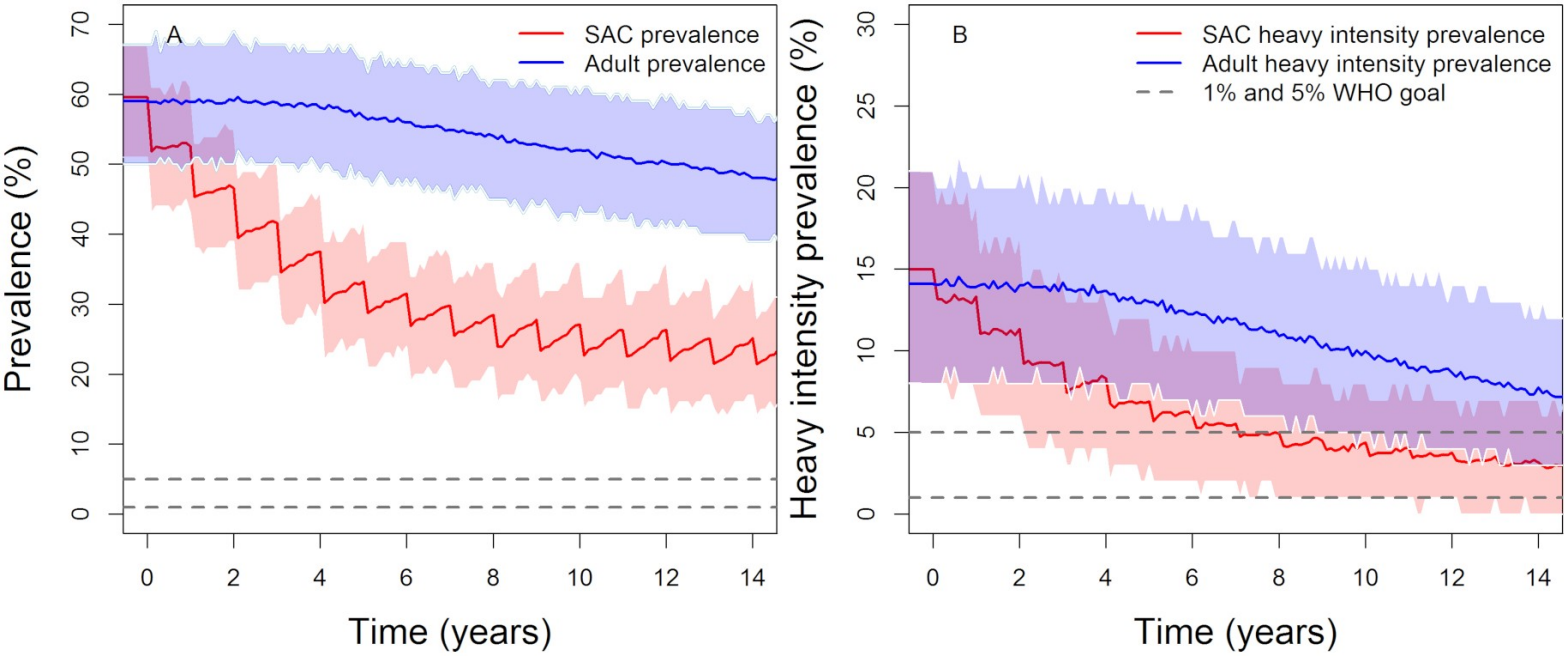
Model projections of annual vaccination in high baseline transmission settings. The duration of vaccine protection is set at 5 years and 1, 6 and 11 years old are vaccinated with a coverage of 85%, 60% and 70% respectively. Graph (A) shows the prevalence of infection, Graph (B) shows the prevalence of heavy-intensity infections, both in school-aged children (SAC) and adults. The average duration of vaccine protection is set at 5 years. Shaded areas (both blue and red) represent the 90% credible interval (90% of the simulated results fall within these shaded areas).

If the duration of vaccine protection is lowered to an average of 5 years, to compensate for this short duration, it is necessary to increase coverage levels to achieve morbidity control or elimination. In the analyses presented in Table 6 and Fig 5, the ages at which individuals are vaccinated, and their coverage levels, are assumed to be: (i) vaccinate at age 1, 6, and 11 years with coverage levels 85%, 60% and 70%, respectively or (ii) vaccinate at age 5, 10, 15 years with coverage levels 60%, 70% and 45%, respectively. These treatment strategies (of vaccinating three different age groups each year—such that in the longer-term individuals will receive more than one dose to maintain protection) produce better results than when we consider a vaccine of protection duration of 20 years. This is because we are treating more age groups when the vaccine has a shorter duration of protection than when it has a longer protection. However, more vaccine is being used in these strategies employing a short duration of protection vaccine.

**Table 6:** Probability of reaching the WHO 1% elimination as a public health problem goal and the 5% SAC 5% morbidity goal for vaccination only with different coverage levels. The duration of protection is set at 5 years. Green shaded areas = probability of reaching target > 90%, Yellow shaded areas = 0.5 ≤ probability of reaching target < 0.9, Red shaded areas = probability of reaching target <0.5.

Transmission setting	Coverage	Probability of achieving morbidity control (≤5% heavy infection in SAC)			Probability of achieving elimination as a public health problem (≤1% heavy infection in SAC)		
		Year 5	Year 10	Year 15	Year 5	Year 10	Year 15
**Low**	85% age 1, 60% age 6, 70% age 11	1	1	1	0.910	0.980	0.993
	60% age 5, 70% age 10, 45% age 15	1	1	1	0.943	0.970	1
**Moderate**	85% age 1, 60% age 6, 70% age 11	0.943	1	1	0.357	0.783	0.953
	60% age 5, 70% age 10, 45% age 15	0.940	1	1	0.380	0.793	0.940
**High**	85% age 1, 60% age 6, 70% age 11	0.300	0.720	0.890	0.000	0.100	0.220
	60% age 5, 70% age 10, 45% age 15	0.430	0.690	0.800	0.002	0.080	0.180

At first sight this result suggests that the duration of protection is not the key parameter in controlling or eliminating morbidity if vaccine cost, supply and delivery are not limiting factors. However, the duration of protection will be a key factor when it is difficult to achieve high coverage in practice, or if cost is a key factor in the management of the control programme. Cost effectiveness/benefit analyses will be key in the situation where the vaccine only offers a short duration of protection.

Coverage levels and probabilities of achieving WHO goals are given in Table 6. For the two treatment strategies the simulations results indicate the following:

- In low transmission settings, the SAC 5% morbidity and 1% elimination as a public health problem goals are achieved within 5 years of treatment with a probability of almost 1.
- In moderate transmission settings, the SAC 5% morbidity goal is achieved within 5 years of treatment, while it takes 15 years for the elimination as a public health problem goal to be achieved.
- In high transmission settings the 1% elimination as a public health problem cannot be achieved, but the SAC 5% morbidity goal can be achieved with a probability of 0.89, when we treat 1,6 and 11 age groups. This probability value is higher than that generated by the MDA only scenario. Vaccination is therefore predicted to be better than MDA in high transmission settings.

In general, the comparisons with the MDA alone control strategy, good coverage of MDA across bands of age classes (i.e. SAC) has a greater and quicker impact than cohort immunization in all settings, even with a long duration of vaccine protection and coverage. This is true because it will take time for herd immunity to develop via the cohort approach and so MDA only will be less efficient in the longer term. In practice, the optimal policy will depend on costs of the vaccine since the MDA drug is donated. MDA costs arise, however, from the logistics of delivery to those who need treatment. The comparison of both costs, MDA and vaccination, will determine what is the most desirable control option.

### MDA and vaccination administrated concurrently

In the previous two sections it is shown that the WHO 5% morbidity control goals can be achieved in low to moderate transmission settings if either MDA alone or vaccination alone are administrated in endemic regions. However, these goals, particularly the 1% elimination as a public health problem goal, are unlikely to be achieved in high transmission settings. Whether it is beneficial to combine both treatments together is examined in this section. In practice, this is a likely scenario since MDA will remain the main control options for many years to come (possibly 10 to 15 years) even if Phase I, II and III trials in humans of the new vaccine go smoothly.

**Fig 6:**
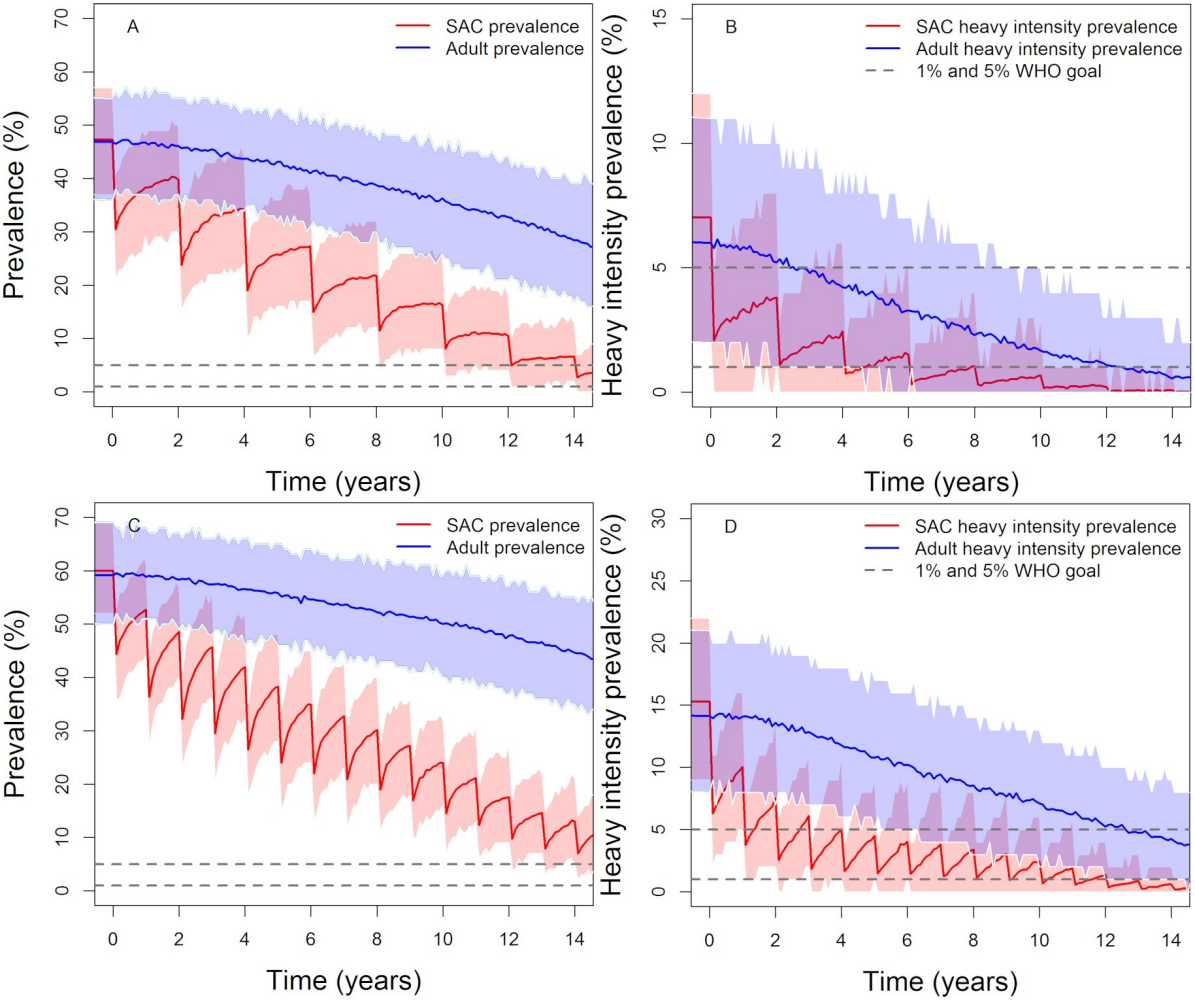
Model projections of vaccination in moderate (first row) and high (second row) baseline transmission settings. The duration of vaccine protection is set at 20 years vaccinating 85% of 1-year olds and giving MDA to 75% of SAC (annually in high transmission settings and once every two years in moderate transmission settings). Graph (A, C), show the prevalence of intensity and Graph (B, D) show the prevalence of heavy-intensity infections in school-aged children (SAC) and adults. Shaded areas (both blue and red) represent the 90% credible interval (90% of the simulated results fall within these shaded areas).

**Fig 7:**
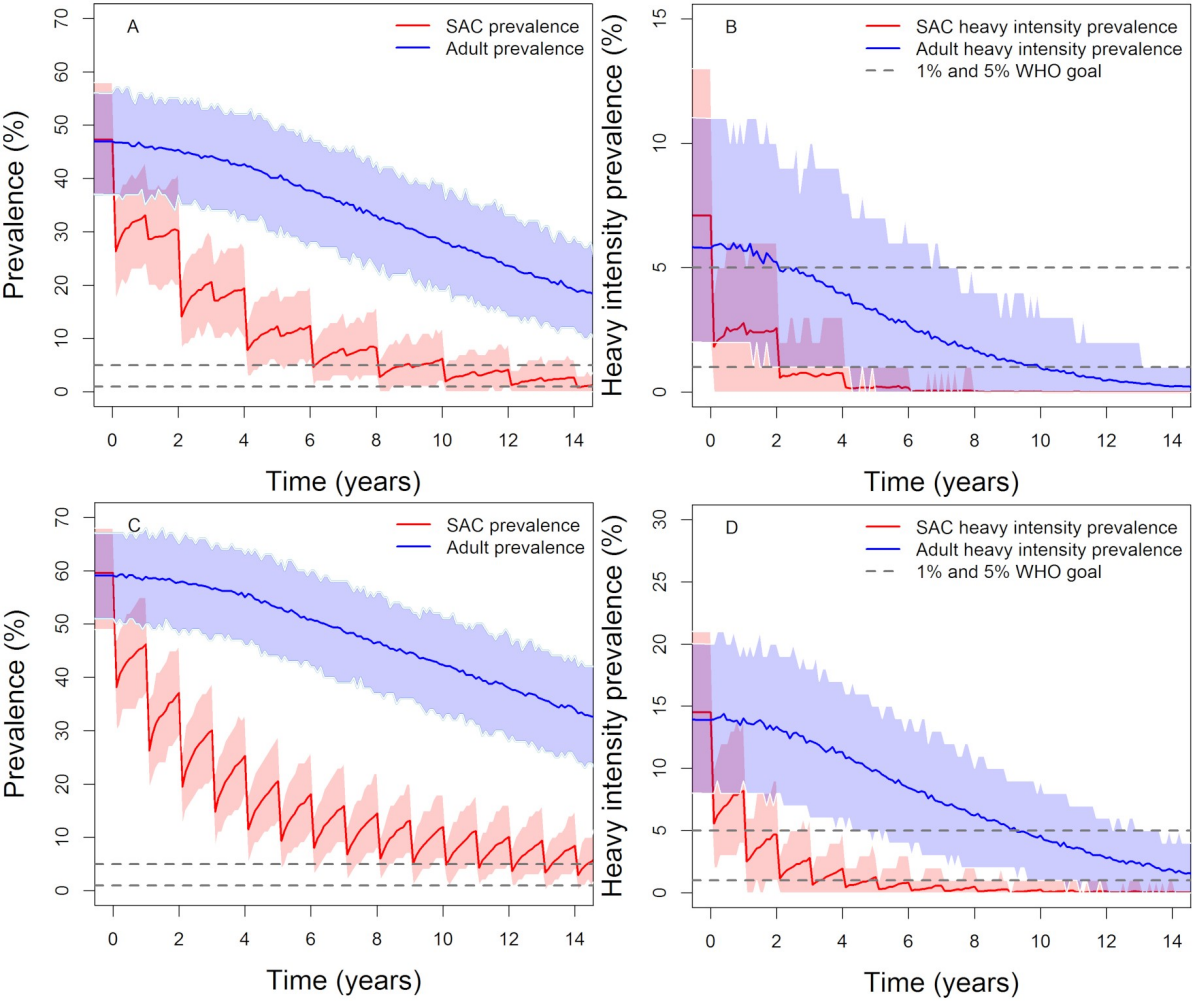
Model projections of vaccination in moderate (first row) and high (second row) baseline transmission settings. The duration of vaccine protection is 5 years, vaccinating 1, 6 and 11-year olds with a coverage of 85%, 60% and 70% respectively, plus MDA given to 75% of SAC (annually in high transmission settings and once every two years in moderate transmission settings). Graphs (A, C), show the prevalence of intensity and Graphs (B, D) show the prevalence of heavy-intensity infections in school-aged children (SAC) and adults. Shaded areas (both blue and red) represent the 90% credible interval (90% of the simulated results fall within these shaded areas).

The simulation results suggest that giving MDA to 75% of SAC and administrating vaccination with a wide range of coverage levels (see Figs 6 and 7, Tables 7 and 8), can reach the 1% elimination as a public health problem goal in high settings with a probability of nearly 0.55 and 0.82 for vaccines with durations of protection of 20 and 5 years, respectively. The 5% SAC morbidity goal is achieved in all transmission settings. Therefore, a vaccine that provides 5 years of protection and covers three age groups, can achieve the WHO 5% morbidity control and 1% elimination as a public health problem goals. However, for a vaccine that provides 20 years protection we need to increase MDA and vaccination coverage levels, or include other age categories in the vaccination programme, to increase the probability of achieving elimination as a public health problem (<1%) in high transmission settings. However, do note that the short duration vaccine must be delivered to multiple age groups. Over 15 years an individual may need three vaccinations (or 3 short courses of vaccination) to maintain protection. As such costs and delivery may be important issues with a short duration of protection vaccine.

**Table 7:** Probability of reaching the WHO 1% elimination as a public health problem goal and the SAC 5% morbidity goals, when MDA and vaccination are administrated concurrently; 75% SAC coverage (annually in high settings, once every 2 years in moderate settings and once every 3 years in low settings) and the vaccine duration of protection is set at 20 years. Green shaded areas = probability of reaching target > 0.9, Yellow shaded areas = 0.5 ≤ probability of reaching target < 0.9, Red shaded areas = probability of reaching target <0.5.

Transmission setting	Coverage	Probability of achieving morbidity control (≤5% heavy infection in SAC)			Probability of achieving elimination as a public health problem (≤1% heavy infection in SAC)		
		Year 5	Year 10	Year 15	Year 5	Year 10	Year 15
**Low**	**85% at age 1**	1	1	1	0.900	0.960	0.993
	**60% at age 5**	0.973	1	1	0.493	0.960	0.993
**Moderate**	**85% at age 1**	0.993	0.997	1	0.720	0.793	1
	**60% at age 5**	0.980	1	1	0.693	0.943	1
**High**	**85% at age 1**	0.170	0.470	0.970	0.007	0.050	0.540
	**60% at age 5**	0.213	0.900	0.973	0.010	0.240	0.580

**Table 8:** MDA, 75% coverage in SAC (annually in high settings, once every 2 years in moderate settings and once every 3 years in low settings) and vaccine duration of protection is set at 5 years. Green shaded areas = probability of reaching target > 90%, Yellow shaded areas = 0.5 ≤ probability of reaching target < 0.9, Red shaded areas = probability of reaching target <0.5.

Transmission setting	Coverage	Probability of achieving morbidity control (≤5% heavy infection in SAC)			Probability of achieving elimination as a public health problem (≤1% heavy infection in SAC)		
		Year 5	Year 10	Year 15	Year 5	Year 10	Year 15
**Low**	85% age 1, 60% age 6, 70% age 11	1	1	1	0.980	1	1
	60% age 5, 70% age 10, 45% age 15	1	1	1	0.987	1	1
**Moderate**	85% age 1, 60% age 6, 70% age 11	1	1	1	0.983	1	1
	60% age 5, 70% age 10, 45% age 15	1	1	1	0.960	1	1
**High**	85% age 1, 60% age 6, 70% age 11	0.890	1	1	0.290	0.700	0.840
	60% age 5, 70% age 10, 45% age 15	0.900	0.983	1	0.290	0.700	0.820

## Sensitivity analysis and model limitations

The results presented in this paper are very sensitive to the values of certain parameters. The two most important are the negative binomial aggregation parameter *k* and the magnitude of transmission before control measures are initiated (the magnitude of R_0_). Using *k* = 0.24, *λ* = 0.24 in low transmission settings, the model cannot support endemic parasite populations when *R*_0_ is low. As a result, the model typically cannot reproduce endemic prevalences less than about 49%. The two possible causes are: (i) Diagnostic; due to poor sensitivity in the standard diagnostic test, measured prevalences may be much lower than the real values and (ii) model transmission structure; transmission may be confined to specific age groups as elimination is approached, giving a low community-level prevalence.

To manage this limitation, we use *k* = 0.04 value for low transmission setting and *k* = 0.24 for moderate to high transmission settings.

We have chosen the extreme baseline prevalences (just below 10% for low transmission settings and just below 50% for moderate transmission settings). For these values there is a high probability to achieve the WHO goals and hence lowering the baseline prevalence does not alter the outcome.

For a baseline prevalence between 50% and 58% (high transmission settings) we obtain qualitatively similar results with the ones produced in moderate settings. Therefore, for high transmission settings, we consider endemic regions with a baseline prevalence of around 62% (*R*_0_ = 3.5) which is a realistic upper bound of prevalence for *S*. *mansoni* in most endemic regions [29], [30]. In this study, we have used parameter values fitted to data collected in Iietune village in Kenya (refer to Table 2), but the same model and analysis can be used for other endemic regions. We should note here, that if the age-related contact rates and death rates are similar to the ones we have used, the results will be similar. If the prevalence of intensity is higher (lower) in SAC, the probability of achieving the WHO goals will be lower (higher) in these regions. These results are based on data for *S*. *mansoni*, but the analysis can be easily extended to *S*. *haematobium*.

A possible key parameter in the analysis and not included in our study is the buildup of acquired immunity. To date, there aren’t enough evidences to show the presence of immunity in *S*. *mansoni* and we have assumed that the shape of age-intensity of infection is influenced only by rate of exposure to infection. It will be of great importance, in the future, to extend our model so that we can explore the effect of acquired immunity on morbidity.

## Discussion

Currently schistosome control strategies suggested by WHO and widely implemented in endemic regions include mass drug administration of school aged children and adults in high transmission settings. The primary goal is morbidity prevention in SAC or morbidity elimination in populations in areas of endemic infection. Snail control, snail habitat alterations and improving water, sanitation and hygiene (WASH) are also recommended (there is little information on their efficacy), but MDA is the major route for morbidity control at present.

In this paper, we have extended the individual based stochastic age structured model developed by Anderson and colleagues, which is constructed on the template of an age structured deterministic model [13–15] where its predictions have been validated using observed infection trends under defined levels of MDA in a number of field settings [31]. We specifically extend past work to include the effect of a vaccine on parasite establishment. The aim has been to explore the impact a vaccine with an efficacy of 100% might have on control efforts to attain the WHO goals for morbidity control in SAC and morbidity elimination in the total population (but not infection). Different treatment and vaccination strategies have been considered in numerical analyses; namely: MDA alone, vaccination alone, or MDA plus vaccination combined. Analyses are conducted for three different transmission settings as defined by WHO on the basis of prevalence; low (<10% baseline prevalence among SAC), moderate (10–50% baseline prevalence among SAC) and high (≥50% baseline prevalence among SAC) settings. These transmission conditions at baseline are determined by the magnitude of *R*_0_, and, concomitantly, by the overall prevalence of infection and the average intensity of infection in defined community.

We find that the optimal strategy to control or eliminate morbidity depends on the transmission setting, vaccine coverage level achieved, the duration of vaccine protection and the timeline of vaccination in different age groupings of the human host.

In low prevalence settings, MDA alone or vaccination alone, with different levels of protection, can achieve the WHO goals with a probability of close to unity. Furthermore, our results show that treating just 40% of SAC with MDA alone can achieve the morbidity control goal and potentially elimination as a public health problem goal. This is an encouraging prediction considering the difficulties endemic regions are having in achieving the WHO recommended treatment coverage for SAC at 75%.

In moderate prevalence settings, treating 60% of MDA can achieve the morbidity goal with probability of unity and possibly the elimination as a public health problem goal with probability of 0.7. Increasing the SAC coverage to 75% increases the probability of elimination to 0.96. Vaccination with a duration of protection of 5 years can achieve the morbidity control goal within 5 years of treatment and elimination as a public health problem goal within 15 years. However, a vaccine with a longer duration of protection (20 years) achieves the morbidity goal with a probability of near unity, but the probability of elimination as a public health problem goal decreases to nearly 0.55.

In high transmission settings, we obtain the following outcomes: (i) the WHO recommended MDA treatment coverage for SAC at 75% can achieve the morbidity control goal with a probability of 0.85, but there is only a 0.35 chance that we can achieve the elimination as a public health problem goal. (ii) Vaccinating 85% of 1-year olds with a vaccine that provides 20 years of protection, can achieve the morbidity control goal with probability of 0.61, but it is very unlikely that the elimination as a public health problem goal will be achieved. (iii) increasing the vaccination coverage levels (vaccinating 85% of age 1, 60% of age 6, 70% of age 11 or vaccinating 60% of age 5, 70% of age 10 and 45% of age 15) and decreasing the duration of protection to 5 years, increases the probability of achieving the WHO goals. For the morbidity control this probability increases from 0.61 to 0.89, while the probability of the elimination goal increases from 0,06 to 0.22.

Thus, in high transmission settings, vaccination alone or MDA alone cannot achieve the elimination as a public health problem target. We can modify this outcome by vaccinating across bands of age classes (i.e. including adults). However, this may risk a high frequency of adverse effects due to past or present infection in vaccinated individuals. The best strategy in these circumstances is intensive MDA plus vaccination. Treating 75% of SAC with MDA and vaccinating 60% of age 5, 70% of age 10 and 45% of age 15 (duration of vaccine protection is 5 years) can achieve the morbidity control goal with probability of unity and the elimination as a public health problem with a probability of nearly 0.84. Alternatively, increasing the SAC coverage to 85% and including 40% of adults in the treatment plan, could achieve the WHO goals with a high probability. This outcome is in line with previous results found in [3], which has reported that including adults in the treatment strategy and increasing SAC coverage levels can lower the prevalence of heavy-intensity to below 1% in SAC.

Analysing the vaccine’s administration schedule (early start versus starting vaccination on entry to school), vaccinating 5-year olds may arguably be an easier strategy to implement than vaccinating 1-year olds. Relatively few individuals will have become infected by 4 years of age, but some have. The main argument in favour of the latter age is that it is easier to reach children for vaccination via school infrastructure/attendance. Alternatively, if the vaccine is safe for very young children (< 1 year of age) then the vaccine could just be part of the national immunization schedule for infants and young children. The other benefit of vaccinating at age 1 is to avoid morbidity induced by early infection in infancy. Given the long duration of vaccine protection, model simulations suggest little difference between the two strategies. This suggests that programmatic and cost issues will be most important in public health policy formulation for the use of the vaccine.

Comparing vaccination with a long duration of protection and MDA alone, we find that good coverage of MDA across bands of age classes (i.e. SAC) is predicted to have a greater and quicker impact than cohort immunization in all settings. However, we have used different coverage levels between these two treatment strategies with less people being vaccinated than are treated with MDA. On the other hand, a vaccine with a shorter duration of protection performs better (in terms of achieving the WHO goals) because we are treating more age groups.

Unless true elimination of transmission is achieved, treatment should not cease as there is a chance that the prevalence of infection will bounce back after cessation. True elimination is not achieved in any of the scenarios considered. Unless the treatment frequencies and coverage levels are increased considerably from the scenarios examined it is very unlikely that this goal will be achieved.

Factors such as individual adherence to treatment is not taken into consideration and we have assumed a random treatment adherence at each round for a given coverage level. The simulations may therefore be on the optimistic side since a proportion of the chosen individuals for a given coverage are likely to be nonadherent over many rounds of MDA [13], [21], [23], [32], [33]. It will be of great importance to have the relevant adherence data to make more accurate predictions.

The predictions presented in this paper depend on the assumptions made concerning the precise nature of the manner in which the intensity of infection varies by age in a given endemic region, the magnitude of *R*_0_ (= transmission intensity) reflected by the baseline prevalence prior to the introduction of control measures. It will be harder to achieve the WHO targets if infection in the very young (pre-SAC) and adults is high. We have used data for *S*. *mansoni* but the same methods of analysis can be applied for S. *haematobium* infection.

In summary, vaccination alone or in combination with MDA, proves to be an effective method to control or eliminate schistosomiasis as a public health problem. Achievement of the WHO goals for morbidity control and elimination depends on vaccine efficacy, on the duration of vaccine protection and on the coverage levels achieved in different age classes.

## References

[1] WHO | Schistosomiasis: progress report 2001–2011, strategic plan 2012–2020. WHO. World Health Organization; 2017; https://www.who.int/neglected_diseases/resources/9789241503174/en/

[2] WHO | Helminth control in school age children: a guide for managers of control programmes. WHO. World Health Organization; 2016; https://www.who.int/neglected_diseases/resources/9789241548267/en/

[3] Toor J, Alsallaq R, Truscott JE, Turner HC, Werkman M, Gurarie D, et al Are we on our way to achieving the 2020 goals for schistosomiasis morbidity control using current world health organization guidelines? Clin Infect Dis. 2018; 29860290 10.1093/cid/ciy001 PMC5982704

[4] Anderson RM, Turner HC, Farrell SH, Truscott JE. Studies of the Transmission Dynamics, Mathematical Model Development and the Control of Schistosome Parasites by Mass Drug Administration in Human Communities. Adv Parasitol. 2016; 10.1016/bs.apar.2016.06.003 27756455

[5] WHO. Preventive chemotherapy in human helminthiasis. WHO Libr Cat Publ Data. 2006

[6] Montresor A, Garba A. Treatment of preschool children for schistosomiasis. 2017. 10.1016/S2214-109X(17)30202-4 28619212

[7] Toor J, Turner HC, Truscott JE, Werkman M, Phillips AE, Alsallaq R, et al The design of schistosomiasis monitoring and evaluation programmes: The importance of collecting adult data to inform treatment strategies for Schistosoma mansoni. Shiff C, editor. PLoS Negl Trop Dis. Public Library of Science; 2018;12: e0006717 10.1371/journal.pntd.0006717 30296257 PMC6175503

[8] Anderson RM, Turner HC, Farrell SH, Yang J, Truscott JE. What is required in terms of mass drug administration to interrupt the transmission of schistosome parasites in regions of endemic infection? Parasites and Vectors. 2015; 10.1186/s13071-015-1157-y 26489831 PMC4618750

[9] WHO | PCT databank. WHO. World Health Organization; 2018; http://www.who.int/neglected_diseases/preventive_chemotherapy/sch/en/

[10] Zhang W, Molehin AJ, Rojo JU, Sudduth J, Ganapathy PK, Kim E, et al Sm-p80-based schistosomiasis vaccine: double-blind preclinical trial in baboons demonstrates comprehensive prophylactic and parasite transmission-blocking efficacy. Ann N Y Acad Sci. 2018;1425: 38–51. 10.1111/nyas.13942 30133707 PMC6110104

[11] Stylianou A, Hadjichrysanthou C, Truscott JE, Anderson RM. Developing a mathematical model for the evaluation of the potential impact of a partially efficacious vaccine on the transmission dynamics of Schistosoma mansoni in human communities. Parasit Vectors. 2017;10: 294 10.1186/s13071-017-2227-0 28623957 PMC5474049

[12] King CH. The evolving schistosomiasis agenda 2007–2017—Why we are moving beyond morbidity control toward elimination of transmission. PLoS Negl Trop Dis. 2017; 10.1371/journal.pntd.0005517 28426653 PMC5398522

[13] Turner HC, Toor J, Hollingsworth TD, Anderson RM. Economic Evaluations of Mass Drug Administration: The Importance of Economies of Scale and Scope. Clin Infect Dis. 2018;66: 1298–1303. 10.1093/cid/cix1001 29126255 PMC5888956

[14] Anderson RM, May RM (Robert M. Infectious diseases of humans : dynamics and control [Internet]. Oxford University Press; 1991. https://global.oup.com/academic/product/infectious-diseases-of-humans-9780198540403?cc=gb&lang=en&

[15] Anderson RM, May RM. Helminth Infections of Humans: Mathematical Models, Population Dynamics, and Control. Adv Parasitol. 1985; 10.1016/S0065-308X(08)60561-8 3904343

[16] Truscott JE, Turner HC, Farrell SH, Anderson RM. Soil-Transmitted Helminths. Advances in parasitology. 2016 pp. 133–198. 10.1016/bs.apar.2016.08.002 27756454

[17] Cheever AW. A Quantitative Post-Mortem Study of Schistosomiasis Mansoni in Man. Am J Trop Med Hyg. The American Society of Tropical Medicine and Hygiene; 1968;17: 38–64. 10.4269/ajtmh.1968.17.38 5637021

[18] Tingley GA, Butterworth AE, Anderson RM, Kariuki HC, Koech D, Mugambi M, et al Predisposition of humans to infection with Schistosoma mansoni: evidence from the reinfection of individuals following chemotherapy. Trans R Soc Trop Med Hyg. 1988;82: 448–52. Available: http://www.ncbi.nlm.nih.gov/pubmed/3148233 3148233 10.1016/0035-9203(88)90159-9

[19] Wright JE, Werkman M, Dunn JC, Anderson RM. Current epidemiological evidence for predisposition to high or low intensity human helminth infection: a systematic review. Parasit Vectors. BioMed Central; 2018;11: 65 10.1186/s13071-018-2656-4 29382360 PMC5791198

[20] Bärenbold O, Raso G, Coulibaly JT, N’Goran EK, Utzinger J, Vounatsou P. Estimating sensitivity of the Kato-Katz technique for the diagnosis of Schistosoma mansoni and hookworm in relation to infection intensity. French M, editor. PLoS Negl Trop Dis. Public Library of Science; 2017;11: e0005953 10.1371/journal.pntd.0005953 28976979 PMC5643140

[21] Fulford AJ, Butterworth AE, Ouma JH, Sturrock RF. A statistical approach to schistosome population dynamics and estimation of the life-span of Schistosoma mansoni in man. Parasitology. 1995;110 (Pt 3): 307–16. Available: http://www.ncbi.nlm.nih.gov/pubmed/7724238 7724238 10.1017/s0031182000080896

[22] Zwang J, Olliaro PL. Clinical Efficacy and Tolerability of Praziquantel for Intestinal and Urinary Schistosomiasis—A Meta-analysis of Comparative and Non-comparative Clinical Trials. Jones MK, editor. PLoS Negl Trop Dis. 2014;8: e3286 10.1371/journal.pntd.0003286 25412105 PMC4238982

[23] WHO Expert Committee on the Control of Schistosomiasis. Prevention and control of schistosomiasis and soil-transmitted helminthiasis: report of a WHO expert committee. World Health Organization; 2002. 12592987

[24] Haddison EC, Abdullahi LH, Muloiwa R, Hussey GD, Kagina BM. Comparison of school based and supplemental vaccination strategies in the delivery of vaccines to 5–19 year olds in Africa—a systematic review. F1000Research. Faculty of 1000 Ltd; 2017;6: 1833 10.12688/f1000research.12804.1 29375814 PMC5765397

[25] Black E, Richmond R. Prevention of Cervical Cancer in Sub-Saharan Africa: The Advantages and Challenges of HPV Vaccination. Vaccines. Multidisciplinary Digital Publishing Institute (MDPI); 2018;6 10.3390/vaccines6030061 30205561 PMC6161067

[26] Bruni L, Diaz M, Barrionuevo-Rosas L, Herrero R, Bray F, Bosch FX, et al Global estimates of human papillomavirus vaccination coverage by region and income level: a pooled analysis. Lancet Glob Heal. 2016;4: e453–e463. 10.1016/S2214-109X(16)30099-7 27340003

[27] WHO | Immunization coverage. WHO. World Health Organization; 2019; https://www.who.int/immunization/monitoring_surveillance/routine/coverage/en/

[28] Anderson R, Farrell S, Turner H, Walson J, Donnelly CA, Truscott J. Assessing the interruption of the transmission of human helminths with mass drug administration alone: optimizing the design of cluster randomized trials. Parasit Vectors. BioMed Central; 2017;10: 93 10.1186/s13071-017-1979-x 28212667 PMC5316156

[29] Shen Y, King CH, Binder S, Zhang F, Whalen CC, Evan Secor W, et al Protocol and baseline data for a multi-year cohort study of the effects of different mass drug treatment approaches on functional morbidities from schistosomiasis in four African countries. BMC Infect Dis. BioMed Central; 2017;17: 652 10.1186/s12879-017-2738-5 28962552 PMC5622450

[30] Sa I, Kamal W, Hk S. Gr up SM Schistosoma Prevalence World-Wide [Internet]. 2016. www.smgebooks.com

[31] De Vlas SJ, Gryseels B, Van Oortmarssen GJ, Polderman AM, Habbema JD. A model for variations in single and repeated egg counts in Schistosoma mansoni infections. Parasitology. 1992;104 (Pt 3): 451–60. Available: http://www.ncbi.nlm.nih.gov/pubmed/1641245 1641245 10.1017/s003118200006371x

[32] Shuford K V., Turner HC, Anderson RM. Compliance with anthelmintic treatment in the neglected tropical diseases control programmes: a systematic review. Parasit Vectors. 2016;9: 29 10.1186/s13071-016-1311-1 26813098 PMC4729159

[33] Babu B V., Babu GR. Coverage of, and compliance with, mass drug administration under the programme to eliminate lymphatic filariasis in India: a systematic review. Trans R Soc Trop Med Hyg. Narnia; 2014;108: 538–549. 10.1093/trstmh/tru057 24728444

